# Habitat Fragmentation Differentially Affects Genetic Variation, Phenotypic Plasticity and Survival in Populations of a Gypsum Endemic

**DOI:** 10.3389/fpls.2017.00843

**Published:** 2017-05-26

**Authors:** Silvia Matesanz, María Luisa Rubio Teso, Alfredo García-Fernández, Adrián Escudero

**Affiliations:** Área de Biodiversidad y Conservación, Departamento de Biología y Geología, Física y Química Inorgánica, Universidad Rey Juan CarlosMóstoles, Spain

**Keywords:** habitat fragmentation, gypsophile, evolutionary potential, *Centaurea hyssopifolia*, gene flow, phenotypic plasticity, quantitative genetic variation, neutral genetic variation

## Abstract

Habitat fragmentation, i.e., fragment size and isolation, can differentially alter patterns of neutral and quantitative genetic variation, fitness and phenotypic plasticity of plant populations, but their effects have rarely been tested simultaneously. We assessed the combined effects of size and connectivity on these aspects of genetic and phenotypic variation in populations of *Centaurea hyssopifolia*, a narrow endemic gypsophile that previously showed performance differences associated with fragmentation. We grew 111 maternal families sampled from 10 populations that differed in their fragment size and connectivity in a common garden, and characterized quantitative genetic variation, phenotypic plasticity to drought for key functional traits, and plant survival, as a measure of population fitness. We also assessed neutral genetic variation within and among populations using eight microsatellite markers. Although *C. hyssopifolia* is a narrow endemic gypsophile, we found substantial neutral genetic variation and quantitative variation for key functional traits. The partition of genetic variance indicated that a higher proportion of variation was found within populations, which is also consistent with low population differentiation in molecular markers, functional traits and their plasticity. This, combined with the generally small effect of habitat fragmentation suggests that gene flow among populations is not restricted, despite large differences in fragment size and isolation. Importantly, population’s similarities in genetic variation and plasticity did not reflect the lower survival observed in isolated populations. Overall, our results indicate that, although the species consists of genetically variable populations able to express functional plasticity, such aspects of adaptive potential may not always reflect populations’ survival. Given the differential effects of habitat connectivity on functional traits, genetic variation and fitness, our study highlights the need to shift the focus of fragmentation studies to the mechanisms that regulate connectivity effects, and call for caution on the use of genetic variation and plasticity to forecast population performance.

## Introduction

Quantitative genetic variation within populations is the substrate for phenotypic evolution and as such, it represents a key aspect of their adaptive evolutionary potential. When environmental conditions change, the presence of genetic variation for ecologically important traits may increase their ability to adapt to the new conditions, which may in turn affect not only plant fitness but also population persistence (Jump et al., 2009; Hoffmann and Sgrò, 2011). Therefore, a positive correlation between genetic variation and fitness could be expected in natural populations (Newman and Pilson, 1997; Booy et al., 2000; Leimu et al., 2006). Furthermore, phenotypic plasticity –the ability of individuals to alter their phenotype in response to the environment–can also allow plant populations to accommodate rapid environmental changes, which can maintain or even increase fitness under oncoming conditions (Matesanz et al., 2010 and references therein). Accordingly, populations that are able to express adaptive functional plasticity can be expected to maintain higher fitness (Sultan, 1995; Pigliucci and Schlichting, 1996). In other words, populations with high evolutionary potential and/or plasticity are paradigmatically predicted to have higher fitness. Traditionally, genetic variation and phenotypic plasticity have been considered as mutually exclusive (but see Wright, 1931; Stearns, 1989), but it is now recognized that these aspects of phenotypic diversity can occur simultaneously, with varying degrees of overlap between them across populations (Hoffmann and Merilä, 1999; Berg et al., 2005; Valladares et al., 2014). Although the relationship between genetic variation and fitness has been experimentally established in several cases (Leimu et al., 2006; Jump et al., 2009), studies simultaneously assessing the quantitative genetic variation of key ecological traits and their plasticity as well as fitness –and the interplay among them– across multiple plant populations are virtually non-existent.

Habitat fragmentation decreases the size and increases the isolation of habitat patches, transforming the landscape into a mosaic of fragments of varying size and connectivity. These aspects of habitat fragmentation –fragment size and connectivity– may have an impact on the amount and distribution of genetic variation of plant populations, and ultimately, on their fitness (Ellstrand and Elam, 1993; Young et al., 1996; Aguilar et al., 2006; Leimu et al., 2006; Willi et al., 2006). Accordingly, the size and connectivity of habitat fragments may affect the expression of phenotypic plasticity at the population level if reduced genetic variation associated with the fragmentation process is also correlated with loss of norms of reaction diversity (Matesanz and Valladares, 2014), although this hypothesis has rarely been tested. A great majority of fragmentation studies considers the effects of fragment size to be driven only by changes in population size (see e.g., Bowman et al., 2008; Gonzalez-Varo et al., 2010), and as such, the effects of both fragment and population size are often confounded. However, fragment size *per se* may have an effect on plant populations independently of population size. For instance, if pollinators are less attracted to small habitat patches (Dauber et al., 2010), populations in small and isolated fragments (even of moderate size) may have reduced genetic variation, plasticity and fitness due to restricted gene flow and increased mating among relatives (Young et al., 1996; Leimu et al., 2006, 2010). Estimates of genetic variation and population differentiation using neutral molecular markers are useful to detect potential gene flow restrictions, the effects of genetic drift and the existence of inbreeding (Kirk and Freeland, 2011; Avise, 2012) in fragmented populations.

A few studies have separately assessed the effects of different components of habitat fragmentation on fitness (Lienert, 2004; Kolb, 2005), neutral genetic variation (Van Rossum et al., 2004; Gómez-Fernández et al., 2016), quantitative genetic variation (e.g., Weber and Kolb, 2014; Walisch et al., 2015) and phenotypic plasticity (e.g., Berg et al., 2005). However, to our knowledge, no study to date has jointly assessed the effects of two key components of habitat fragmentation such as fragment size and connectivity (and their interaction), on neutral and quantitative genetic variation, phenotypic plasticity and fitness of multiple populations.

Plant species growing on gypsum soils provide a fitting model to assess the effects of habitat fragmentation on genetic variation, plasticity and fitness. In combination with semiarid conditions, gypsum soils give rise to unique habitats that host many endemic and rare species, constituting a remarkable biodiversity hotspot in terrestrial ecosystems (Mota et al., 2011; Escudero et al., 2015). Gypsum habitats are characterized by an island-like configuration due to the discontinuous distribution of gypsum outcrops and the differential establishment of plant species on contrasting vegetation bands within the gypsum islands. Beyond this natural fragmentation, these habitats are subject to human-induced fragmentation associated to agriculture and afforestation practices (Matesanz et al., 2009, 2015). Many of the taxa that inhabit gypsum habitats are gypsophiles, i.e., specialist plants that grow exclusively on gypsum soils. A few authors have argued that edaphic endemics may be evolutionary *dead-ends*, since endemism may result in low genetic variation and therefore preclude evolution (Rajakaruna et al., 2014). Therefore, knowledge on the genetic variation and plasticity of such species in a global change context can be particularly important due to their high specificity for gypsum soils, which can limit their ability to migrate to more suitable habitats as well as their adaptive potential (Mota et al., 2011; Damschen et al., 2012; Escudero et al., 2015).

In this study, we evaluated neutral and quantitative genetic variation, phenotypic plasticity and a major fitness component (survival) on populations of the gypsophile *Centaurea hyssopifolia* Vahl. (Compositae) that differ on their fragment size and connectivity. Previous studies have shown that habitat fragmentation has detrimental effects on multiple reproductive fitness traits in this species, both in natural and controlled conditions (Matesanz et al., 2009, 2015; Pias et al., 2010). However, it is not yet known whether habitat fragmentation reduces neutral and quantitative genetic variation, and/or phenotypic plasticity in this species, and whether these are reflected by fitness responses. Using maternal families from 10 populations originating from fragments along independent gradients of size and connectivity, we measured a combination of morphological and physiological traits as well as fitness in a common garden experiment. Furthermore, to assess populations’ responses to drought, we applied two contrasting watering treatments and assessed plasticity patterns for these traits. Finally, we used eight microsatellite markers to provide a measurement of neutral genetic variation within and among populations. We addressed the following specific questions: (i) Do populations of the gypsophile *C. hyssopifolia* have differing levels of within-population neutral genetic diversity? (ii) Do populations show quantitative genetic variation and phenotypic plasticity for ecologically important functional traits, and do they vary across populations? (iii) If so, is population differentiation related to fragment size and connectivity?, and (iv) Is survival correlated with the amount of genetic variation and plasticity in each population?

## Materials and Methods

### Study Species and Source Populations

*Centaurea hyssopifolia* Vahl. (Compositae) is a dominant species of the dwarf shrub community on Iberian gypsum soils, restricted to gypsum massive soils of central Spain (Luzuriaga et al., 2006). The species is a medium-lived, small chamaephyte (20–60 cm) that exclusively grows on gypsum outcrops, i.e., it is a genuine gypsophile. It occurs on the upper piedmont of gypsum slopes, where a sparse shrub community of gypsophiles establishes. Long-term field observations suggest that individuals of *C. hyssopifolia* have an average longevity of 4–10 years. Flowering spans from May to July, with inflorescences (capitula) presenting purplish disk florets and a few white ray florets. The species is described as a mainly outcrosser with partial self-compatibility (Luzuriaga et al., 2006), which concurs with the mating system of other close congeners, and has limited dispersal mechanisms, with achenes with almost no pappus. Preliminary field data revealed that individually bagged inflorescences produced no viable seeds (Luzuriaga et al., unpublished). Field pollinator censuses showed that the study species is visited by a taxonomically diverse group of pollinators, including Bombyliidae species, several Apidae species as well as Lepidoptera and Coleoptera (unpublished data).

Populations were sampled in a fragmented gypsum landscape in the Tajo River Basin, near Chinchón, central Spain. Long-term, intensive agricultural activities have progressively fragmented the landscape, creating a mosaic of patches of natural vegetation remnants interspersed in a matrix of dry herbaceous croplands (cereal), dry arboreal croplands (olive trees), vineyards and *Pinus halepensis* plantations. Climate is semiarid Mediterranean with mean annual rainfall of 422 mm and mean temperature of 13.8°C. The study fragments laid within a 5 km × 6 km rectangular area (600–700 m a.s.l.), so climatic conditions across sites were very similar. We selected 10 populations from fragments that differed markedly in their size and degree of connectivity (see full details of fragment selection in Matesanz et al., 2015). We used high-resolution orthophotos of the area to identify and digitize remnant patches of gypsum vegetation (≈300 fragments). We measured the area of each fragment and calculated the minimum distance among surrounding fragments. To quantify the connectivity of each fragment (i.e., inverse of isolation), we used an index that accounts for the number of surrounding fragments weighed by their distance to the target fragment and their size (Tremlova and Muenzbergova, 2007). Specifically, connectivity was expressed as:

$$C_{i}=\log_{10}\sum_{k=1}^{n}A_{k}/d_{ik}^{2},i\neq k$$

where *C*_i_ (unitless) is the connectivity of fragment *i, n* is the total number of natural fragments around the target fragment that are included within a 500 m radius circle, *A*_k_ is the area of fragment *k*, and *d*_i_ is the minimum distance between fragment *i* and *k* (see Supplementary Table S1 for distance among selected fragments). We used a conservative radius of 500 m because pollen movement among fragments located at larger distances is likely to be minimal, since generalist pollinators can forage pollen from many sources within a single fragment (Aizen et al., 2002; Fontaine et al., 2008). This index was selected because it takes into account not only the number of fragments of natural habitat surrounding the target fragment but also the among-fragment distance and their size, providing a complete measure of landscape connectivity. Based on these preliminary data, we surveyed 100 fragments in the field and finally selected 10 fragments of contrasting size and connectivity based on the following criteria: (i) the study species was abundant, with at least 150 individuals in each fragment, to evaluate the effects of fragment size *per se*, irrespective of population size and (ii) there was a well-developed biological soil crust, since its presence is indicative of unmanaged, undisturbed gypsum habitats. We selected fragments whose age (earlier date at which the fragment was identified in historical aerial photographs; **Table 1**) was much higher than the generation time of the species. The selected fragments create both a size and a connectivity gradient. Fragment size ranged from 1241 to 138849 m^2^ (100-fold difference), and fragment connectivity ranged from 1.83 to 6.97 (**Table 1**). In order to test the independent effects of both components of habitat fragmentation –size and connectivity– as well as their interaction, we ensured that the gradients were not correlated (non-significant correlation between fragment size and connectivity *R* = 0.12, *P* = 0.76).

**Table 1:** Fragment description.

Fragment code	Latitude	Longitude	Fragment size (Ha)	Fragment connectivity	Minimum age (date)	Aspect	Altitude (m)
5	40°9′14.40″	3°28′9.50″	1.6	4.506	1946	SE	630
6	40°9′10.20″	3°28′19.00″	4.84	6.415	1956	S	618
119	40°9′11.10″	3°28′29.40″	0.12	5.001	1956	SE	618
121	40°9′25.80″	3°28′44.40″	0.82	6.968	1956	S	600
133	40°7′20.60″	3°29′9.50″	3.52	3.249	1956	SE	662
136	40°7′22.10″	3°29′38.60″	0.12	1.826	1956	S	669
139	40°7′13.20″	3°28′20.20″	13.88	3.741	1975	SW	667
250	40°10′14.70″	3°25′31.80″	9.2	5.436	1946	NE	696
255	40° 9′0.20″	3°26′8.90″	6.09	4.036	1975	NW	712
302	40°7′16.8″	3°28′48.37″	0.45	2.111	1946	S	633

*Location (geographic coordinates), size, connectivity, age of fragment, aspect and altitude for the 10 fragments sampled. Minimum age refers to the earliest date when the fragment was visible based on historical aerial photographs. See text and Matesanz et al. (2015) for details on fragment selection.*

At the peak of the reproductive season in 2012, we randomly selected 12 reproductive individuals located within a 20 m × 20 m plot that was established in the south or south–east slope of each fragment, to minimize microclimatic differences among plots (**Table 1**). Plants were located ≥1 m from each other and were chosen to represent a random sample of the plant size distribution in each fragment. We collected 50–70 mature capitula from each mother plant (hereafter family). Capitula were dissected and viable seeds were cleaned and stored. In total, we sampled 120 families from 10 fragments. Since the sampled maternal families were open-pollinated, plants from the same family are considered half-siblings (Falconer and Mackay, 1996). Fresh leaves were also collected from 20 plants per fragment (including the maternal plants), air dried and stored in paper bags.

### Molecular Markers

Neutral genetic variation was estimated using eight microsatellite markers transferred from other species of the genus *Centaurea* (Fréville et al., 2000; Marrs et al., 2006; Austin et al., 2011; see Supplementary Table S2). DNA was extracted from 60 mg of leaf tissue using the SpeedTools Plant DNA Extraction kit (Biotools, Madrid, Spain), following the manufacturer protocol. PCR conditions for each microsatellite marker are detailed in Supplementary Table S1. PCR products were verified in an agarose 1% gel stained with 1% of RedGel (Biotium, Fremont, CA, United States) and amplified in an ABI 3730 (Applied Biosystems, Madrid, Spain) in Unidad de Genómica (Universidad Complutense, Madrid, Spain). Scoring was performed using GeneMarker v. 2.61 (SoftGenetics, State College, PA, United States).

### Experimental Sample, Common Garden, and Plasticity to Drought Experiment

Five viable seeds per plant were randomly selected and weighed individually in a Mettler Toledo MX5 microbalance (1 μg precision; Mettler Toledo, Columbus, OH, United States), to assess family mean seed mass. In October 2012, simulating germination following natural dispersal, seeds of each maternal family were randomly selected and sown in common conditions in an experimental glasshouse at the CULTIVE facilities at URJC (690 m a.s.l). Seeds were sown in 1.4 L pots (10 cm × 10 cm × 17 cm) filled with locally collected topsoil and topped with a 4 cm layer of sieved gypsum substrate. This gypsum layer was collected from a single patch at the study site by removing the top layer of substrate to avoid the naturally occurring seed bank, and was added to improve germination. Ten pots per family were included in the experiment, and seven seeds were sown in each pot. During the germination period, pots were periodically watered to ensure permanent humidity, and temperature in the experiment was controlled to match the optimum reported for this species. The 10 pots with plants from the same family were randomly assigned to each of five blocks (two pots per maternal family; 240 pots per block) and arranged in a randomized complete block design. After 5 weeks, when no significant new emergence was observed, emerged seedlings were clipped to leave one plant per pot. Due to low germination of a few maternal families of some populations and several missing replicates, the final sample was 1099 plants (9–12 families/fragment × 10 fragments × 10 replicates). See Supplementary Table S3 for details on number of families and plants per fragment.

All plants were grown for ≈1 year under favorable conditions of water and nutrient availability, and several functional traits as well as survival were measured during this period to assess genetic variation and population differentiation (see below). After this period, we performed the plasticity experiment. We assessed plasticity to drought because water is the most limiting environmental factor in the Mediterranean; indeed, drought has been shown to be the main cause of seedling mortality in natural conditions in other coexisting gypsophiles (e.g., Escudero et al., 2000). In August 2013, simulating a typical Mediterranean summer drought, half of the remaining plants were assigned to a drought treatment and the other half was maintained in well-watered conditions (total *N* = 727 plants). In the drought treatment, soil moisture was slowly reduced by spacing out watering events (see Supplementary Figure S1). Soil moisture (expressed as % of field capacity for this substrate) was monitored weekly by weighing a random sample of 15 pots in each watering treatment. Plasticity to drought (details on measurements below) was evaluated when plants experienced a reduction of ≈50% of field capacity. After this, irrigation of plants in the drought treatment was ceased until all plants died.

### Data Collection

#### Common Conditions

Pots were monitored weekly for 5 weeks after sowing to record emergence (both cotyledons observed above soil level). Emergence rate was calculated as the number of seedlings emerged during this period divided by the number of seeds sown in each pot (seven). Survival (as a measure of fitness) of each plant was recorded throughout the experiment, either fortnightly or monthly. Rosette size for each plant was estimated as the area of an ellipse by measuring the maximum diameter (*d*_1_) and a second perpendicular diameter (*d*_2_), as π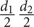. Rosette size was estimated one (December 2012) and 6 months (May 2013) after emergence. In May 2013, leaf number was measured in all plants. Relative growth rate was estimated for all plants as RGR = (Ln S_2_ – Ln S_1_)/T_2-1_, where S_1_ and S_2_ are plant size at time 1 and 2, respectively, and T_2-1_ is the time elapsed between the two measurement dates. In June 2013, we measured leaf length in two leaves of all plants using a digital caliper (0.01 mm resolution), and collected one fully developed and healthy leaf from 3 to 4 replicates per family. We measured leaf size (area) of collected leaves in a portable area meter Li-3000C (Li-Cor, NE, United States). Leaves were then oven-dried for 48 h at 65°C and weighed. Specific leaf area (SLA) was estimated as the ratio of the one-side area of a fresh leaf divided by its oven-dry mass (Cornelissen et al., 2003). Chlorophyll fluorescence (photochemical efficiency, *F*_v_/*F*_m_) was measured with a portable pulse-modulated fluorometer (FMS2, Hansatech, United Kingdom) from 9 to 12 am in one leaf previously adapted to dark for 30 min with leaf clips. Minimal (*F*_o_) and maximal (*F*_m_) fluorescence were measured, and these values were used to calculate photochemical efficiency (*F*_v_/*F*_m_) as *F*_v_/*F*_m_ = (*F*_m_ -*F*_o_)/*F*_m_, where *F*_v_ is the difference between *F*_m_ and *F*_o_. In July 2013, plant height was measured in all plants, and leaf thickness was measured in one leaf from one replicate per family using a dial thickness gauge (Mitutoyo Co., Aurora, IL, United States). Finally, stomatal conductance was measured from 9 to 12 am in one replicate per family with a leaf porometer (SC-1, Decagon Devices, Pullman, WA, United States).

#### Plasticity to Drought Experiment

Survival of each plant was monitored since the onset of the drought treatment until all plants died (January 2014). Furthermore, a set of morphological and physiological traits were measured in all surviving plants (1–4 replicates per family and treatment) when soil moisture in the drought treatment was 40–50% of field capacity (mid-September-early October 2013, see Supplementary Figure S1). We measured leaf number and height, leaf size, SLA, photochemical efficiency and stomatal conductance in all plants as described above.

### Data Analyses

#### Neutral Genetic Diversity

For each population, we estimated the following genetic diversity indices: *A*, average number of alleles per locus and per population; *H*_o_, observed heterozygosity (number of heterozygotes/N, where *N* is the number of individuals per population) and *H*_E_, gene diversity or Nei’s unbiased expected heterozygosity [(2N/(2N-1)) ^∗^ (1-Σpi2), where pi is the frequency of the ith allele for the population], using the programs GenAlEx v. 6.501 (Peakall and Smouse, 2006) and Genepop v. 4.2 (Rousset, 2008). The inbreeding coefficient, *F*_IS_, was estimated using INEst 2.0 software (Chybicki and Burczyk, 2009) that corrected for the excess of homozygosity due to the effects of null alleles and genotyping errors [using 50 × 10^5^ Markov Chain Monte Carlo (MCMC) iterations, burn-in = 50000 and thinning = 50]. To determine population differentiation in molecular markers, we computed pairwise *F*_ST_ (Weir and Cockerman, 1984) with *P*-values for each pair of populations using FSTAT (Goudet, 1995).

#### Population Differentiation in Quantitative Traits

We used linear mixed models with restricted maximum likelihood (REML) to test for the fixed effects of fragment size, fragment connectivity, their interaction, and block on each phenotypic trait. Population identity (i.e., fragment identity) and Family nested within population were included as random factors (variance components), and family mean seed mass as a covariate. Seed mass was included as a proxy of maternal effects, as it is commonly used as a measure of maternal investment (Roach and Wulff, 1987; Wulff et al., 1994; Lacey et al., 1997). Including population identity in the models as a random factor is a conservative approach, since part of the variability among fragments is probably due to the fixed factors, i.e., the random factor could be absorbing part of the effects of fragment size and connectivity. Analyses were performed using package lme4 in R. Significance of fixed effects was assessed via *F*-tests with type III error (using the function “Anova” of library “car”). Significance of variance components was tested by likelihood ratio tests, by comparing the full model (including fixed and random factors) with the reduced model (dropping the random factor; (Zuur et al., 2009).

#### Quantitative Genetics Parameters

Variance components were extracted from each model (described above) to calculate the following quantitative genetic parameters. Narrow-sense heritability was calculated as:

$$h^{2}=\frac{\sigma_{A}^{2}}{\sigma_{ph}^{2}}=\frac{4\sigma_{f}^{2}}{\sigma_{p}^{2}+\sigma_{f}^{2}+\sigma_{e}^{2}}$$

where $\sigma_{f}^{2}$ represents the family genetic variance, $\sigma_{\mathrm{ph}}^{2}$ is the total phenotypic variance, $\sigma_{p}^{2}$ is the phenotypic variance explained by differences among populations and $\sigma_{e}^{2}$ is the residual variance. The additive genetic variance ($\sigma_{A}^{2}$) was estimated as 4 × $\sigma_{f}^{2}$, as we assumed that each offspring of a dam (maternal plant) has a different sire (pollen donor). If multiple ovules from the same plant were pollinated by the same pollen donor, then the relatedness of the offspring (replicate siblings from a given family) from the same plant would be greater than is assumed here, and the estimates of additive genetic variance would be upwardly biased (Falconer and Mackay, 1996; Santiso et al., 2015). Ninety-five percentage confidence intervals for the heritability estimates were computed using a bootstrapping approach with 1000 simulations, using the function “bootMer” in package lme4. We also calculated the coefficient of genetic variation (CVA) as √$\sigma_{A}^{2}$ divided by the trait mean (Houle, 1992), for traits with values only at one side of zero. This parameter provides a measure of genetic variation standardized by the mean, allowing to qualitatively comparing genetic variation among different traits (e.g., Agrawal et al., 2008; Santiso et al., 2015). Finally, to provide an index of genetically based variance, we additionally examined the proportion of phenotypic variance attributed to differences among families, as $\sigma_{f}^{2}$/$\sigma_{\mathrm{ph}}^{2}$, and to differences among populations, as $\sigma_{p}^{2}$/$\sigma_{\mathrm{ph}}^{2}$ [see (Facon et al., 2008; Matesanz et al., 2014) for other studies using these metrics as estimates of genetic variation]. The proportion of phenotypic variance explained by differences among families was also calculated for each population and trait separately, and was extracted from models testing for the effect of family performed for each population. The latter was used as a measure of quantitative genetic variation within each population.

#### Plasticity to Drought

To assess the plastic response to the water treatments, mixed models with REML were performed testing for the fixed effects of water treatment (well-watered vs. drought), and block and the random effect of population and the interaction of population by water treatment. Family was not included in these models due to low replication within families associated with mortality along the experiment. Significance of each term of the model was assessed as described above. A significant treatment effect is evidence for plasticity in the trait. A significant population × treatment interaction means that there is genetic variation for plasticity among the study populations. This interaction signifies the evolutionary potential for plasticity at the species level (Matesanz and Valladares, 2014). Furthermore, indices of phenotypic plasticity were calculated as the percentage of change in the mean trait value from one environment to the other as P = (
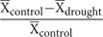
) × 100, where *P* is plasticity, *X*_control_ is the mean trait value for each population in the control (well-watered) treatment and *X*_drought_ is the mean trait value for each population under drought conditions (Valladares et al., 2006). We used the Kaplan–Meier method to estimate differences in survival between watering treatments and among populations in response to drought. Survival analyses were performed in STATISTICA 8 (Statsoft, Tulsa, OK, United States).

#### Effects of Habitat Fragmentation

We used linear models to assess the effects of populations’ characteristics, i.e., fragment size and connectivity, and their interaction, on the indices of neutral genetic variation and quantitative genetic variance within populations (% of total phenotypic variance associated to family differences). These tests were repeated using the raw variance estimate (instead of the percentage) associated to the factor Family, to correct for differences among populations in total phenotypic variance, which gave very similar results. Linear models were also used to test the effects of fragment size, connectivity and their interaction on plasticity indices. The Kaplan–Meier method was used to calculate cumulative survival curves of families from each population. Differences among populations in mortality rate and dynamics were tested using the log-rank test. Finally, Pearson correlations were used to determine the relationship between genetic variation (neutral and quantitative), phenotypic plasticity and population fitness (estimated as final survival). Correlations were performed in STATISTICA 8 (Tulsa, OK, United States).

## Results

### Neutral Genetic Variation within and among Populations

Genetic diversity was high in all populations (**Table 2**). The eight microsatellite loci scored gave a total of 130 alleles in the 199 individuals of *C. hyssopifolia* successfully amplified, with an average of 16.25 alleles per locus. All loci were polymorphic with 4–33 alleles per locus. The average number of alleles observed per locus ranged from 6.30 in population *136* to 8.50 in population *133* (**Table 2**). Expected heterozygosity values were generally high and did not vary much among populations (from 0.61 to 0.67; **Table 2**). Observed heterozygosity ranged from 0.52 to 0.69 and was slightly lower than expected heterozygosity (**Table 2**). The inbreeding coefficient (*F*_IS_) was very low, ranging from 0.02 to 0.09, and not significantly different from zero (**Table 2**). Population pairwise *F*_ST_ values were low yet significantly different from zero, with values ranging from 0.0113 to 0.104 (Supplementary Table S4).

**Table 2:** Genetic diversity indices of the 10 populations of *Centaurea hyssopifolia* sampled.

	*N*	*A*	*H*_o_	*H*_E_	*F*_IS_
5	19	7.50	0.52	0.65	0.07 (3)
6	20	6.63	0.57	0.64	0.04 (2)
119	20	7.25	0.59	0.66	0.04 (2)
121	20	7.38	0.55	0.66	0.04 (2)
133	20	8.50	0.61	0.65	0.07 (1)
136	20	6.13	0.60	0.61	0.02 (1)
139	20	7.25	0.58	0.67	0.07 (2)
250	20	7.13	0.60	0.65	0.07 (1)
25	20	7.50	0.56	0.64	0.05 (2)
302	20	7.50	0.69	0.65	0.09 (3)
Overall	199	7.28	0.59	0.65	0.06

*N number of individuals sampled, *A* average number of alleles per locus, *H*_*o*_ observed heterozygosity, *H*_*E*_ expected heterozygosity, *F*_*IS*_ inbreeding coefficient; number of loci showing significant deviations from the Hardy–Weinberg equilibrium are in parenthesis.*

### Quantitative Genetic Variation within and among Populations

All the measured plant traits (except for height, SLA and chlorophyll fluorescence) showed demonstrable genetic variance (significant Family term in **Table 3**), indicating that these traits have the potential to respond to selection. Narrow-sense heritability was variable, ranging from 0.119 (chlorophyll fluorescence, *F*_v_/*F*_m_) to 0.934 (emergence rate), and was significantly different from zero in most cases (entire confidence intervals above zero; **Table 4**). Similarly, the coefficient of genetic variance (CVA) and the percentage of phenotypic variance explained by family differences were lower for chlorophyll fluorescence and larger for emergence rate (**Table 4**). When evaluated for each population, we found no consistent pattern for the amount of genetic variance within populations. All populations had significant genetic variation for several traits (significant Family term, Supplementary Figure S2), but the percentage of variance explained by the factor Family varied widely across traits and populations.

**Table 3:** Results from Restricted Maximum Likelihood models testing the fixed effects of fragment size, connectivity, their interaction and block, and the random effects of fragment and family (nested within fragment) on the study traits.

	Fragment size	Fragment connectivity	Size × Connectivity	Block	Seed mass	Population	Family (population)
Emergence rate	0.577	1.731	1.056	0.243	2.584^†^	5.446***	142.641***
Rosette size (1 m)	0.155	0.013	0.000	3.519**	4.331^∗^	25.757***	47.584***
Rosette size (6 m)	0.412	0.006	0.150	10.730***	0.464	16.454***	45.159***
RGR	0.068	0.016	0.121	9.391***	2.831^†^	53.260***	10.425**
Plant height	0.647	3.659	0.649	6.176***	0.823	1.216	3.127^†^
Leaf number	0.615	1.603	0.016	2.003^†^	1.928	8.877**	44.496***
Leaf length	0.171	1.369	0.315	10.886***	3.701^∗^	8.315**	7.183**
Leaf area	0.170	0.045	0.331	-	0.613	5.016*	20.214***
SLA	1.541	0.458	0.700	-	0.402	3.443^†^	2.720^†^
*F*_v_/*F*_m_	1.362	2.927	1.729	-	1.047	0.379	0.240
Leaf thickness	0.225	0.300	0.307	-	0.051	1.077	-
Stomatal conductance	0.254	0.049	0.092	-	2.377	1.33	-

*Family-mean seed mass was used as a covariate in the models. *F* and χ^*2*^-values for fixed and random factors, respectively, and significance levels are shown for each trait. ^†^*P* < 0.10, ^∗^*P* < 0.05, ^∗∗^*P* < 0.01, ^∗∗∗^*P* < 0.001. The significance of random factors was assessed by comparing the full model with models dropping each random factor.*

**Table 4:** Quantitative genetics parameters for 111 families from 10 populations of *C. hyssopifolia*.

	*h^2^*	95% CI for *h^2^*	CVA	$\sigma_{A}^{2}$	% of phenotypic variance
Emergence rate	**0.934**	(0.646, 1.245)	70.097	0.051	23.356
Rosette size (1 m)	**0.498**	(0.310, 0.727)	27.531	0.522	12.442
Rosette size (6 m)	**0.477**	(0.276, 0.699)	30.955	0.677	11.920
RGR	**0.169**	(0.044, 0.338)	NA	7.34E-6	4.660
Plant height	0.157	(0, 0.366)	21.080	13.513	3.930
Leaf number	**0.496**	(0.288, 0.718)	31.971	5.373	12.400
Leaf length	**0.287**	(0.064, 0.585)	21.699	33.565	7.185
SLA	0.334	(0, 0.827)	16.103	635.514	8.356
Leaf area	**0.936**	(0.474, 1.410)	41.408	0.061	23.389
Chlorophyll fluorescence	0.119	(0, 0.621)	2.117	2.72E-4	2.967

*Narrow-sense heritability, confidence intervals of heritability, coefficient of genetic variation (CVA = √$\sigma_{A}^{2}$/mean), additive genetic variance (4 × Vf) and % of phenotypic variance explained by family. Bold values indicate a significant effect of family in the models (*P* < 0.05).*

**FIGURE 1:**
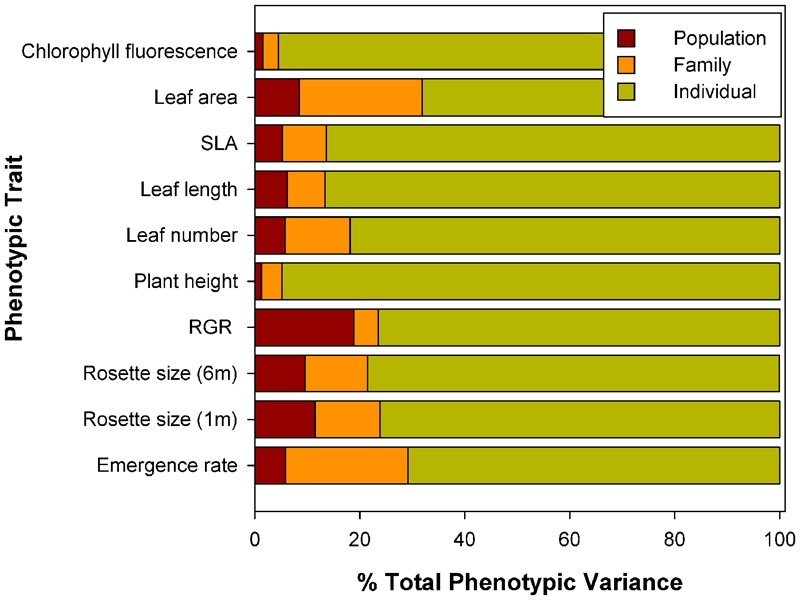
**Percentage of total phenotypic variance attributed to differences among populations, among maternal families within populations, and among individuals within maternal families for a range of growth rates, physiological traits, and morphological traits of *Centaurea hyssopifolia***.

There was also significant genetic variation among populations, i.e., population differentiation, for most traits (significant Population term, **Table 3** and see Supplementary Table S5 for population means). However, differences among families within populations were higher than differences among populations (except for RGR), i.e., differences among families explained a larger proportion of phenotypic variance (range 3–23.4%) than differences among populations (range 1.3–18.9%), and this was very consistent among traits. For all traits, however, the great majority of the variance was explained by differences among individuals (range 68.2–95.5%; **Figure 1**).

### Plasticity to Drought

**FIGURE 2:**
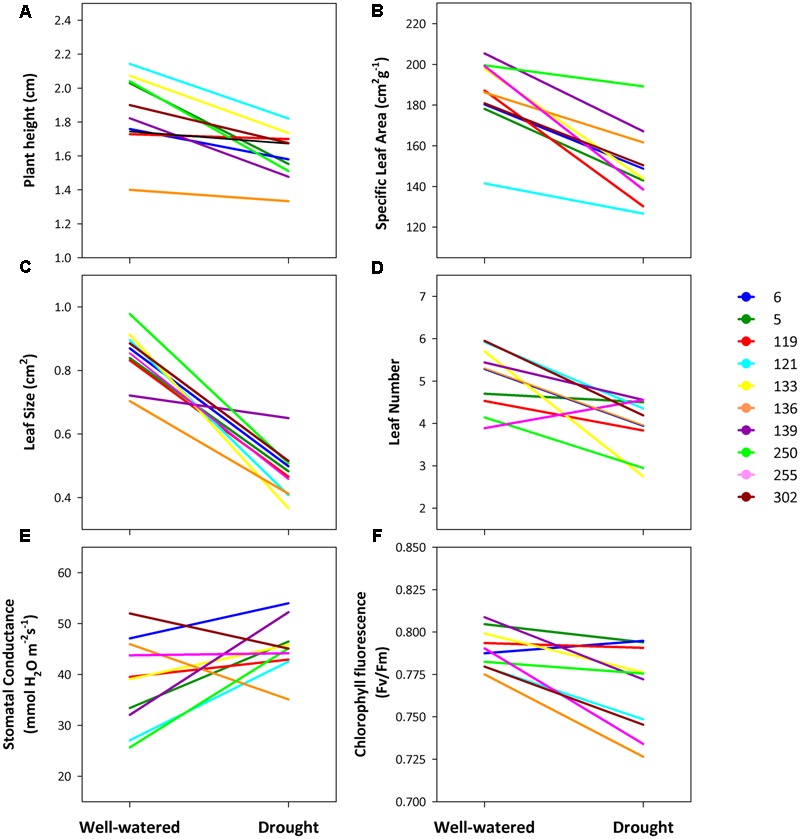
**Norms of reaction for 10 populations of *C. hyssopifolia* at two contrasting watering treatments. (A)** Plant height; **(B)** specific leaf area (SLA); **(C)** leaf size; **(D)**, leaf number, **(E)**, Stomatal conductance and **(F)**, chlorophyll fluorescence (*F*_v_/*F*_m_). Population color codes correspond to those in **Table 1**. See text for details on water treatments and measurements.

The drought treatment exerted a significant effect on all traits except for stomatal conductance (significant Water Treatment term, **Table 5**). Plants from all populations significantly decreased leaf size (78% reduction) and number (28%), SLA (24%), plant height (16%) and chlorophyll fluorescence (5%) in the drought treatment (**Figure 2**). This plastic response was similar across populations (non-significant Population × Treatment interaction, **Table 5**), i.e., there was no population differentiation for plasticity. Drought also had a significant effect on plant survival: plants in dry conditions died faster than those that were under well-watered conditions (log-rank test statistic = -5.20, *P* < 0.0001, df = 1; Supplementary Figure S3). Again, this response was similar for all populations: there were no significant differences among populations in survival in the drought treatment (χ^2^ = 6.87, *P* = 0.65; df = 9).

**Table 5:** Plasticity to drought in populations of *C. hyssopifolia*.

	Water treatment	Block	Seed mass	Population	Population × Treatment
Leaf number	14.951^∗∗^	0.277	2.2859	0	0.373
SLA	27.694^∗∗∗^	**-**	0.0035	1.405^†^	0.046
Leaf size	71.607^∗∗∗^	**-**	0.0001	0	0
Stomatal conductance	3.223^†^	**-**	0.6755	0	1.686
Chlorophyll fluorescence	11.190^∗∗^	**-**	0.8401	1.119	0.053
Plant height	6.947^∗^	2.5801^∗^	0.0000	0.023	0

*Restricted Maximum Likelihood models tested the fixed effects of water treatment and block, and the random effect of population (fragment ID) and the interaction of population with treatment. ^†^*P* < 0.10, ^∗^*P* < 0.05, ^∗∗^*P* < 0.01, ^∗∗∗^*P* < 0.001.*

### Effects of Habitat Fragmentation

**FIGURE 3:**
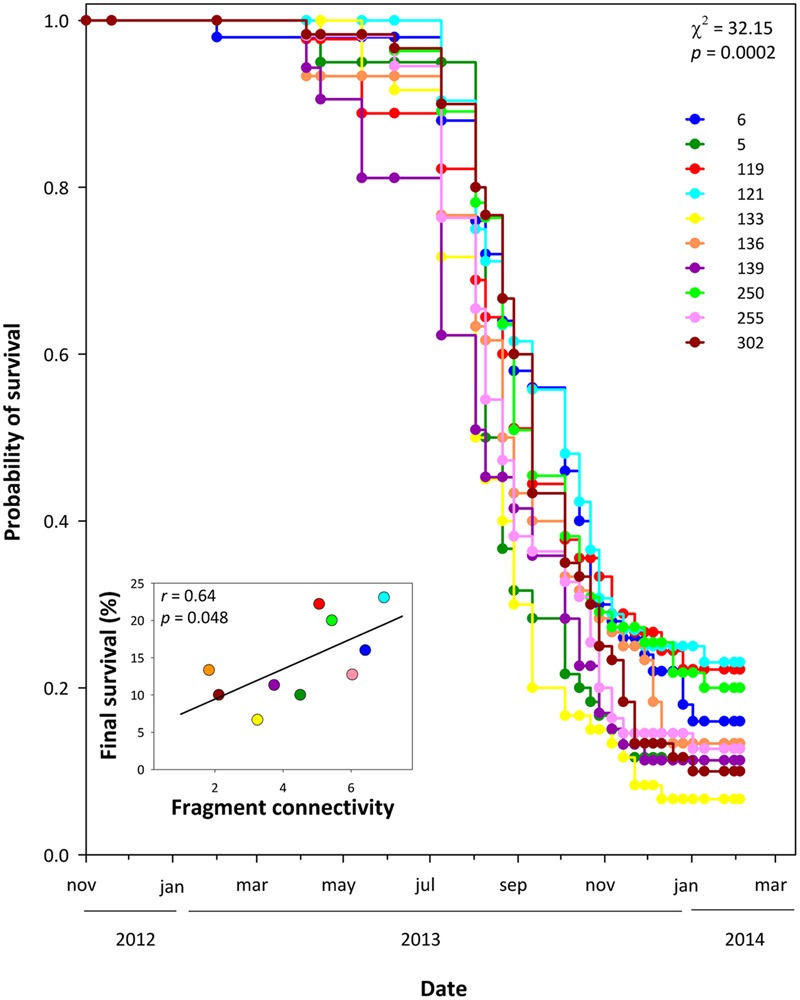
**Survival curves for all populations in well-watered conditions.** Kaplan–Meier curves show cumulative survival at each timepoint. Curves were calculated based on 90–120 plants per population. Inset: relationship between fragment connectivity and percentage of surviving plants at the end of the experiment. Numbers in the legend refer to population codes on **Table 1**.

There was no significant effect of fragment size on the genetic diversity indices per population (Supplementary Table S6). Similarly, there was no significant effect of fragment size and connectivity, or their interaction, on functional traits, i.e., the observed phenotypic differentiation among populations was not related to the fragment characteristics of each population (**Table 3**). Furthermore, we found no effect of fragment size, fragment connectivity or their interaction on the amount of genetic variation (% of phenotypic variance explained by family differences) in each population, or in the expression of phenotypic plasticity, i.e., populations from small/isolated fragments did not show lower genetic variation or less plasticity than populations from large/connected ones (Supplementary Tables S7, S8). However, there was a significant difference in survival among populations that was related to fragment connectivity. Plants from populations from more connected fragments had higher survival than those from populations from isolated fragments (χ^2^ = 32.15, *P* < 0.001, df = 9; **Figure 3**).

### Correlation between Genetic Variation, Plasticity, and Survival

We found no significant correlation between neutral and quantitative genetic variation (% of phenotypic variance averaged for all traits) and population fitness (survival; *r* = 0.173, *P* = 0.633; Supplementary Table S9). Similarly, we found no relationship between phenotypic plasticity (averaged for all traits) and survival (*r* = 0.004, *P* = 0.992) or between plasticity and genetic variation (*r* = -0.324, *P* = 0.368). See Supplementary Table S7 for tests at the trait level.

### Seed Mass Effects

Except for two traits, no significant seed mass effects were detected in functional traits, population differentiation or the expression of plasticity (not significant seed mass term, **Tables 3, 5**).

## Discussion

### Genetic Variation, Phenotypic Plasticity, and Survival in Populations of *Centaurea hyssopifolia*

Populations of the study species from a fragmented landscape showed high neutral genetic variation (comparable to other congeneric species; see, e.g., Fréville et al., 2001; Marrs et al., 2008), and revealed significant genetic variation in life-history, morphological and physiological traits. Furthermore, plants from all populations responded plastically to water stress –the most limiting environmental factor in this system– in patterns consistent with adaptive functional plasticity. Despite large similarities among populations in the amount of neutral and quantitative genetic variation and on the expression of phenotypic plasticity, populations significantly differed in a major fitness component, survival.

Several studies have shown a positive relationship between genetic variation – either neutral and/or quantitative – and plant fitness components (e.g., Hansson and Westerberg, 2002; Leimu et al., 2010). For instance, in populations with low genetic variation, increased inbreeding and allele loss may lead to an increased proportion of homozygotes and the expression of deleterious alleles, which may eventually reduce plant survival (Young et al., 1996; Leimu et al., 2010). Therefore, populations with high genetic variation are expected to also have high fitness. Similarly, it is also an accepted paradigm that (adaptive) phenotypic plasticity has a positive impact on plant fitness (Sultan, 1995; Pigliucci and Schlichting, 1996; Matesanz and Valladares, 2014), and a few studies have experimentally confirmed this expectation (e.g., Lazaro-Nogal et al., 2015). However, in our study, populations showed similar levels of neutral and quantitative genetic variation, and broadly similar patterns of functional plasticity, but significantly differed in their survival. In other words, the population’s genetic variation and evolutionary potential of important functional traits (and their plasticity) were not related to population fitness. Altogether, and contrary to expectations, these results suggest that genetic variation –either molecular or quantitative– and phenotypic plasticity may be in some instances poor predictors of population survival, and are in agreement with other studies where correlations between quantitative genetic variation and various components of plant fitness were not found (Lauterbach et al., 2011; Walisch et al., 2015). More generally, our findings call for caution on the use of standing genetic variation and/or plasticity, as is usually done in biological conservation contexts, to forecast the future performance of populations.

The high genetic variation observed for most functional traits indicate that this gypsum specialist has substantial evolutionary potential, a first requirement to adapt to shifting environmental conditions. Our estimates of narrow-sense heritabilities largely varied among traits (0.119–0.934) but were moderate (<0.5) for 8 of the 10 traits measured (average *h^2^*: 0.44), and are in line with those reported for other species from fragmented landscapes (Mannouris and Byers, 2013; Weber and Kolb, 2014; Ye et al., 2014; Walisch et al., 2015). Lack of significant genetic variation for key traits such as plant height and chlorophyll fluorescence may be due to several factors, including low phenotypic variation due to the species rosette growth form and the lability of ecophysiological traits (see e.g., Agrawal et al., 2008). Only for two traits (emergence rate and leaf area) did we obtain very high values. It has been argued that in open-pollinated plants from mainly self-incompatible plants, a small proportion of the offspring can share the same father, leading to families being composed of both full- and half-siblings, which could bias the estimation of heritability (Falconer and Mackay, 1996). However, the generally high observed heterozygosity observed in these populations together with the fact that only two of 10 traits showed such a pattern suggests that high relatedness among siblings may not be playing a significant role in our calculations, and that outcrossing is indeed the main reproductive mechanism in our populations. Maternal effects can also lead to higher phenotypic similarity among siblings, particularly during early-life stages (Roach and Wulff, 1987). In our study, most of the functional traits were measured more than 6 months after germination, and seed mass effects were negligible in most cases. This suggests that maternal effects are likely to be minor in our study, and supports that the phenotypic differences observed have a genetic basis. We note, however, that unmeasured maternal effects may still have been present on traits expressed very early in life.

Alongside the high within-population genetic variation, plants from all populations responded adaptively to water stress. In addition to the passive response that reflects resource limitation, which included reduced plant size and photosynthetic performance, plants from all populations responded actively to water shortage (a reduction of 50% of soil field capacity) by reducing leaf size as well as the structure of the leaf tissue (SLA). The latter morphological and physiological adjustments are well-understood responses to moisture-limited conditions (Sultan and Bazzaz, 1993; Matesanz and Valladares, 2014). These results contribute to the limited current understanding on the ability of restricted edaphic endemics to express adaptive plasticity to varying environmental conditions (Damschen et al., 2012).

### Effects of Habitat Fragmentation

We did not find any effect of habitat fragmentation, i.e., fragment size and connectivity, on neutral and/or quantitative genetic variation of the study populations. All populations, irrespective of the size and degree of isolation of their source fragment, showed similar levels of neutral genetic variation and had significant quantitative variation – estimated by genetic differences among families– for several functional traits. In other words, populations from small and isolated fragments contained similar amounts of neutral and quantitative genetic variation, i.e., evolutionary potential, than populations from large and connected ones. Similarly, there was no effect of fragment size or connectivity on the measured functional traits or their plasticity.

Despite the lack of effects of habitat fragmentation on trait means, genetic variation and plasticity, we found a significant effect of fragment connectivity on a major fitness component, i.e., survival. Plants from populations from more connected fragments had higher survival in well-watered conditions, which can have important consequences for the long-term viability of populations in isolated sites. Low plant fitness in plants from isolated fragments can be the result of high inbreeding (Lienert, 2004; Kolb, 2005), the fixation of deleterious alleles (Young et al., 1996), seed provisioning (Stanton, 1984) and/or maternal effects (Roach and Wulff, 1987). Inbreeding and deleterious genetic effects seem unlikely in our case due to the low inbreeding coefficients and the high genetic variation observed. Seed provisioning and other maternal effects may have an impact on offspring survival. Matesanz et al. (2015) found lower seed mass on plants from small and isolated fragments. However, the almost negligible effect of seed mass on functional traits, genetic variation and plasticity together with the fact that significant mortality only started when plants were older than one year makes it unlikely that these factors are responsible for differences in survival among populations. Irrespective of the underlying mechanism, our results contribute to the mounting evidence that both fragment size and connectivity have an effect on fitness of *C. hyssopifolia*, either on its survival or reproductive output (Matesanz et al., 2009; Pias et al., 2010; Matesanz et al., 2015).

Lack of fragmentation effects on genetic variation, functional traits and plasticity is unexpected for several reasons. First, a comprehensive meta-analysis showed that self-incompatible species are more susceptible to the detrimental effects of habitat fragmentation than self-compatible ones because of their dependence on pollinator mutualisms (Aguilar et al., 2006). Second, recent studies reported high vulnerability (e.g., lower survival and reproductive output) of *C. hyssopifolia* populations to different components of habitat fragmentation (Matesanz et al., 2009, 2015; Pias et al., 2010). In particular, a field assessment of the fitness effects of fragment size and connectivity performed in the same study site showed lower reproductive output (i.e., fewer viable seeds per capitulum and lower seed set) of plants from isolated fragments (Matesanz et al., 2015), which could lead to lower genetic variation if fewer offspring are contributed to the next generation, and thus effective population sizes decrease over time. Finally, it could be expected that the anatomical features of *C. hyssopifolia* seeds, with a short pappus compared to seed weight, would limit their dispersal ability (Sheldon and Burrows, 1973), in turn decreasing gene flow and increasing the effects of genetic drift (Ellstrand and Elam, 1993; Young et al., 1996; Leimu et al., 2006).

Contrary to these expectations, our results therefore suggest that gene flow –either via pollen or seeds– among *Centaurea* populations is sufficient to maintain similar levels of genetic variation, even in the more isolated fragments. This is consistent with the low population differentiation observed in functional traits, which was in most cases lower than the relatively high within-population variation (**Figure 1**). Unrestricted gene flow is also supported by generally low inbreeding coefficients and low population differentiation estimated with microsatellite markers, indicating that in these populations, conspecific pollen from various sources may be readily available and breeding among relatives may be rare. Altogether, these results suggest that, at the fine scale, the sampled fragments of this species might functionally constitute a metapopulation with similarly connected nodes of different sizes and isolation levels (Manel et al., 2003). Alternatively, although the generation time of the study species is much shorter than the time since the fragments originated, we cannot rule out the possibility that the effects of fragmentation on neutral or quantitative genetic variation will be observed in the long-term (Debinski and Holt, 2000; Ellmer et al., 2011; Mona et al., 2014). Indeed, a lag effect of habitat fragmentation has been observed in several plant and animal species (e.g., Reitalu et al., 2009). Furthermore, various mechanisms other than gene flow, such as overdominance, balancing selection or mutation rates can be responsible for the similar genetic variation observed across populations. A few studies have reported a significant relationship between population size and quantitative variation (Willi et al., 2006). For instance, Weber and Kolb (2014) found higher heritabilities of phenological and reproductive traits in larger populations of the perennial herb *Phyteuma spicatum*. In contrast, our results concur with other studies where no relationship between genetic variation and population and/or fragment size was detected (see e.g., Ellmer et al., 2011; Walisch et al., 2015).

Similarly, there was no effect of fragment size or connectivity on the measured functional traits or their plasticity. Size, growth rate and leaf traits were similar in plants from all populations. Phenotypic differentiation among populations can be the result of differential selective pressures in each population, the effect of neutral processes such as genetic drift and inbreeding, or a combination of both (Merilä and Crnokrak, 2001; Holderegger et al., 2006). For instance, Walisch et al. (2015), in a study of the rare *Saxifraga rosacea* subsp. *sponhemica* from fragmented populations, found that most population trait means were significantly related to climate gradients, suggesting adaptive genetic differentiation in quantitative traits (see also Weber and Kolb, 2014; Ye et al., 2014). Likewise, Kittelson et al. (2015) found that inbreeding significantly affected the expression of 12 functional traits in populations of the prairie forb *Echinacea angustifolia*, and Mannouris and Byers (2013) found a significant effect of genetic drift load on populations of *Chamaecrista fasciculata* from small fragments. In our study, lack of population differentiation in functional traits suggests a low impact of genetic drift and inbreeding on plant traits, and/or similar selective pressures acting on our populations due to similar microclimatic conditions (see also Ellmer et al., 2011). We cannot discard, however, the possibility that populations have adaptively diverged to their local environment in a different set of unmeasured functional traits, or that inbreeding effects will be expressed in more stressful field conditions (Keller and Waller, 2002).

Lack of population differentiation in plasticity patterns may be due to past selection on specific norms of reaction, or to absence of within-population genetic variation for plasticity to evolve (Matesanz et al., 2010). Irrespective of the underlying mechanism, parallel populations’ norms of reaction suggest that fragment size and connectivity had no effect on the expression of phenotypic plasticity to varying water conditions. Our results concur with a study of *Carlina vulgaris* from populations of different size and degree of isolation that found no evidence that small or isolated populations were less plastic than large or connected ones (Berg et al., 2005 but see Kery et al., 2000; Paschke et al., 2005). More generally, these results contribute to our understanding on the yet poorly explored effects of habitat fragmentation on plasticity.

## Conclusion

Our most outstanding finding is the lack of correlation between phenotypic plasticity, genetic variation and survival. Although it is well known that plasticity has an important adaptive value and a strong genetic basis, our results suggest that these aspects of variation can respond to different factors and that they may not be necessarily connected with key fitness components. We found significant evolutionary potential and phenotypic plasticity for key functional traits in populations of *C. hyssopifolia*, which can at least partly contribute to their future persistence and reduce extinction risk (Jump et al., 2009; Hoffmann and Sgrò, 2011). In a global change context, the presence of genetic variation and the ability to express adaptive plasticity can be particularly beneficial for edaphic specialists such as the model species, since their intimate association to a specific soil type, i.e., gypsum, has been suggested as a barrier to face oncoming conditions due to their limited ability to migrate (Damschen et al., 2012; Escudero et al., 2015). The occurrence of both high genetic variation and phenotypic plasticity support the notion that these are not mutually exclusive alternatives, and that both can be present in plant populations. The lack of genetic effects of habitat fragmentation at the fine (landscape) scale suggest that future studies on the impacts of fragmentation should assess population differentiation and genetic isolation at broader spatial scales, using different theoretical frameworks (Manel et al., 2003; McRae and Beier, 2007). Furthermore, our results challenge the link between the evolution of edaphic endemism and genetic variation, and show that such endemics may not necessarily always be evolutionary *dead-ends* (discussed in Rajakaruna et al., 2014; see also see also Martínez-Nieto et al., 2013; Salmerón-Sánchez et al., 2014 for other studies assessing genetic variation in gypsum species). Finally, given the differential effects of habitat connectivity on functional traits (and plasticity), genetic variation and survival and reproductive fitness, our study also highlights the need to shift the focus of fragmentation studies to the mechanisms that regulate connectivity effects at different spatial scales and their interaction with fragment size.

## Author Contributions

SM and AE conceived the study. SM, MLRT, and AG-F collected data. SM analyzed data and wrote the manuscript, with input from all other authors.

## Conflict of Interest Statement

The authors declare that the research was conducted in the absence of any commercial or financial relationships that could be construed as a potential conflict of interest.
