# Supplementary material for: Habitat Fragmentation Differentially Affects Genetic Variation, Phenotypic Plasticity and Survival in Populations of a Gypsum Endemic
Source: Front Plant Sci. 2017 May 26;8:843. doi: 10.3389/fpls.2017.00843 (PMC5445106; doi:10.3389/fpls.2017.00843)
Supplement: Supplementary file 1 [file Data_Sheet_1.docx]

**Supplementary information for Habitat fragmentation differentially affects genetic variation, phenotypic plasticity and survival in populations of a gypsum endemic**

**Table S1.** Distance (in m) among the selected fragments. See Matesanz et al. 2015 (Figure 1) for a map of the area.

|  | 5 | 6 | 119 | 121 | 133 | 136 | 139 | 250 | 255 |
| --- | --- | --- | --- | --- | --- | --- | --- | --- | --- |
| 5 | 0.00 |  |  |  |  |  |  |  |  |
| 6 | 107.98 | 0.00 |  |  |  |  |  |  |  |
| 119 | 358.53 | 8.19 | 0.00 |  |  |  |  |  |  |
| 121 | 636.11 | 418.32 | 413.05 | 0.00 |  |  |  |  |  |
| 133 | 3663.03 | 3294.30 | 3462.89 | 3800.10 | 0.00 |  |  |  |  |
| 136 | 3945.24 | 3529.78 | 3686.28 | 3949.19 | 597.67 | 0.00 |  |  |  |
| 139 | 3501.06 | 3210.12 | 3397.44 | 3803.34 | 732.14 | 1472.78 | 0.00 |  |  |
| 250 | 3458.85 | 4001.16 | 4236.76 | 4297.27 | 7007.54 | 7498.43 | 6295.95 | 0.00 |  |
| 255 | 2349.12 | 2820.75 | 3088.01 | 3357.02 | 4972.11 | 5570.62 | 4060.40 | 1998.94 | 0.00 |
| 302 | 3584.85 | 3122.42 | 3592.27 | 3830.45 | 396.00 | 993.67 | 327.67 | 6611.54 | 4576.11 |

**Table S2**. Transferability process of microsatellite markers from other species of the genus *Centaurea* to *Centaurea hyssopifolia.*

After the search performed in GenBank to evaluate the existence of microsatellite markers in other species of the genus Centaurea, we selected 25 microsatellite loci from four different *Centaurea* species (Table 1). Amplifications were carried out using DNA extracted from 60 mg of tissue using the SpeedTools Plant DNA Extraction kit (Biotools), according the manufacturer protocol. PCR reactions for each locus were done in a 20.2 µL volume containing 14.2 µL of pure Milli-Q water, 2 µL of Buffer with 2 mM MgCl2 (Biotools), 0.2 µL of each dNTP (Biotools), 0.5 µL of each primer and 1 U Taq DNA polymerase (Biotools, Madrid, Spain) and 1.2 µL of template DNA. Amplification reactions were performed in a S1000 Thermal Cycler (Bio-Rad Laboratories, CA, USA) with the following cycle: initial denaturation step of 94 °C for 4 min, followed by 30 cycles of 1 min at 94 °C, 1 min with annealing temperature (Table 1) and 1 min at 72 °C, and a final extension at 72 °C for 7 min. PCR products were verified in an agarose 1% gel stained with 1% of RedGel (Biotium, CA, USA). PCR products were sequenced in a ABI 3730 (Applied Biosystems, Madrid, Spain) in Unidad de Genómica, Universidad Complutense de Madrid (Madrid, Spain) to corroborate the existence of the microsatellite motif. Finally, the study was performed with markers C3, C9, C13, C14, C18, C19, C21 and C25 (Table 1).

**Table S2** (cont). Microsatellite markers used for evaluate the transferability from other *Centaurea* species. * Indicates the microsatellite used for the study.

| Name | Specie | Sequence | Temp of Annealing | GenBank Code | Reference |
| --- | --- | --- | --- | --- | --- |
| C1F | *Centaurea diffusa* | aggtgcactttcctgttcaac | 58 | DQ356426 | Mars et al. 2006 MolEcoNot |
| C1R |  | caacccaataagattacttccacttc |  |  |  |
| C2F | *Centaurea diffusa* | GAAGGGCTACGAGGGTGTTC | 55 | DQ356425 | Mars et al. 2006 MolEcoNot |
| C2R |  | GAAGTGTTGTGCATTTCAATCTATT |  |  |  |
| C3F* | *Centaurea maculosa* | GCTATTAACAACTCCAAAATGAACAG | 45 | DQ356419 | Mars et al. 2006 MolEcoNot |
| C3R* |  | CCTGCTCCAACAAGTTTCCTC |  |  |  |
| C4F | *Centaurea maculosa* | GGCTACATTAAGCTTATCCATTC | 55 | DQ356427 | Mars et al. 2006 MolEcoNot |
| C4R |  | CTCGCATGTTATCCTCCCTC |  |  |  |
| C5F | *Centaurea diffusa* | GGTCCCCATACTTTCAAGCTAATAAC | 55 | DQ356418 | Mars et al. 2006 MolEcoNot |
| C5R |  | ATGCTTCCCTTCTCAATGTTTTCTCT |  |  |  |
| C6F | *Centaurea maculosa* | TGGGATATTCGTTGGTTTAGTTTT | 58 | DQ356422 | Mars et al. 2006 MolEcoNot |
| C6R |  | CCTCCCACTCCCGTTTGAC |  |  |  |
| C7F | *Centaurea maculosa* | GGAGGGCATGGGATTAAAGAGAT | 55 | DQ356424 | Mars et al. 2006 MolEcoNot |
| C7R |  | TGGATGCATCGGTCTGGAAATA |  |  |  |
| C8F | *Centaurea maculosa* | ATGGGACATAAGATCCACAACAG | 45 | DQ356420 | Mars et al. 2006 MolEcoNot |
| C8R |  | TAATTCAGCATTCAAAAATTTAGAAGAC |  |  |  |
| C9F* | *Centaurea maculosa* | TACTTGGGCTTTTCGCTAATGAT | 60 | DQ356421 | Mars et al. 2006 MolEcoNot |
| C9R* |  | ACAAACGTGTTCCAGCAGCAG |  |  |  |
| C10F | *Centaurea maculosa* | gaatgggatgggttcaattatttatg | 55 | DQ356423 | Mars et al. 2006 MolEcoNot |
| C10R |  | atatcaaggggagaagccactatcac |  |  |  |
| C11F | *Centaurea corymbosa* | CATATACACCCACGCACAGC | 62 | AF241184 | Freiville et al. 2000 MolEco |
| C11R |  | GGTGCAGCAAGGAGAGGAC |  |  |  |
| C12F | *Centaurea corymbosa* | GTGCTCCGTCAGCAATCTTT | 62 | AF241182 | Freiville et al. 2000 MolEco |
| C12R |  | GGATGGAGGTGGTGAGGTTA |  |  |  |
| C13F* | *Centaurea corymbosa* | TTTTCAAATATCTTGGTCAAT | 55 | AF241180 | Freiville et al. 2000 MolEco |
| C13R* |  | TGCTGCCATTAATTTTGTCA |  |  |  |
| C14F* | *Centaurea corymbosa* | TTTCTATGCTGTTTGTTTTTGG | 58 | AF241185 | Freiville et al. 2000 MolEco |
| C14R* |  | CCCATACGTCGTCTTCCC |  |  |  |
| C15F | *Centaurea corymbosa* | TGTTAGAAACACAAAAGCATGC | 60 | AF241183 | Freiville et al. 2000 MolEco |
| C15R |  | TTTCCAAAATGAAGTTGAAGGC |  |  |  |
| C16F | *Centaurea corymbosa* | GGAGGCATGCGAACTAAAAG | 62 | AF241181 | Freiville et al. 2000 MolEco |
| C16R |  | CCGGTCTCATGAAAACAACT |  |  |  |
| C17F | *Centaurea corymbosa* | CACACTCACGCTCAGCATTC | 55 | AF241179 | Freiville et al. 2000 MolEco |
| C17R |  | CATCGTTTCCAAACTTCCTC |  |  |  |
| C18F* | *Centaurea aspera* | AACAATGGCGTTCTCATTCAC | 65 | EH716506.1 | Austin et al. 2011 MolEco Res |
| C18R* |  | CGTAGCTAGCCGGAGTCTTTT |  |  |  |
| C19F* | *Centaurea aspera* | ATCTCTCCCATGACCACCTCT | 55,5 | EH754166,1 | Austin et al. 2011 MolEco Res |
| C19R* |  | TCTAGGTTACGGAGACGCTGA |  |  |  |
| C20F | *Centaurea aspera* | GGGGGAGGAAACAACAATAGA | 57 | EH780862.1 | Austin et al. 2011 MolEco Res |
| C20R |  | ATCCACCAAGAACCTCATTCC |  |  |  |
| C21F* | *Centaurea aspera* | GTGGAAGTCGTTGATGGAAGA | 55 | EH783651.1 | Austin et al. 2011 MolEco Res |
| C21R* |  | TCTGTTCCTTTGCATCCATTC |  |  |  |
| C22F | *Centaurea aspera* | CAGTGTGGATGGATGGATACC | 54 | EH789030,1 | Austin et al. 2011 MolEco Res |
| C22R |  | GAAGAATTGCAAAGGGAAAGG |  |  |  |
| C23F | *Centaurea aspera* | AGGACTATTACGCCCCTTTCA | 55,5 | EH751778,1 | Austin et al. 2011 MolEco Res |
| C23R |  | CAGGTCAACTGAGGTTTTGC |  |  |  |
| C24F | *Centaurea aspera* | AAACCATGGAATCAAGTGTCG | 57 | EH777535.1 | Austin et al. 2011 MolEco Res |
| C24R |  | TGAATGCTTGTTTCCCTCATC |  |  |  |
| C25F* | *Centaurea aspera* | GGAGAGTCGGACTGTGAATTG | 55,5 | EH772209.1 | Austin et al. 2011 MolEco Res |
| C25R* |  | ATGCAGCTGTCTCCAATTTGT |  |  |  |

**Table S3**. Number of maternal families and plants per population. The total number of plants in the experiment was 1099.

| **Population** | **Number of families** | **Number of plants** |
| --- | --- | --- |
| 5 | 12 | 120 |
| 6 | 10 | 100 |
| 119 | 9 | 89 |
| 121 | 11 | 105 |
| 133 | 12 | 119 |
| 136 | 12 | 120 |
| 139 | 11 | 106 |
| 250 | 11 | 110 |
| 255 | 11 | 110 |
| 302 | 12 | 120 |

**Table** **S4**. Pairwise *F*_ST_ values for 10 populations of the study species. Significant values are in bold.

|  | 5 | 6 | 119 | 121 | 133 | 136 | 139 | 250 | 255 |
| --- | --- | --- | --- | --- | --- | --- | --- | --- | --- |
| 6 | **0.0359** |  |  |  |  |  |  |  |  |
| 119 | **0.083** | **0.0587** |  |  |  |  |  |  |  |
| 121 | **0.0758** | **0.0533** | **0.0228** |  |  |  |  |  |  |
| 133 | **0.0729** | **0.0444** | **0.0274** | **0.0153** |  |  |  |  |  |
| 136 | **0.1014** | **0.07** | **0.0556** | **0.034** | **0.0303** |  |  |  |  |
| 139 | **0.0603** | **0.0404** | **0.0404** | **0.0509** | **0.0348** | **0.0668** |  |  |  |
| 250 | **0.0634** | **0.0404** | **0.0263** | **0.0176** | **0.0157** | **0.028** | **0.0201** |  |  |
| 255 | **0.0621** | **0.0385** | **0.0388** | **0.0226** | **0.0181** | **0.0266** | **0.0358** | **0.0113** |  |
| 302 | **0.081** | **0.0617** | **0.0358** | **0.0255** | **0.0186** | **0.0243** | **0.0438** | **0.0142** | **0.0194** |

**Table S5**. Morphological, growth and physiological traits (mean ± standard error) of 10 populations of *Centaurea hyssopifolia* from fragments of contrasting size and connectivity.

|  | **Emergence rate** | | **Rosette size (1m)** (cm^2^) | | **Rosette size (6m)** (cm^2^) | | **Relative growth rate** | | **Plant height** (mm) | | **Leaf number** | | **Leaf length** (mm) | | **Leaf area (cm^2^)** | | **SLA** (cm^2^/g) | | **Chlorophyll fluorescence** | |
| --- | --- | --- | --- | --- | --- | --- | --- | --- | --- | --- | --- | --- | --- | --- | --- | --- | --- | --- | --- | --- |
| **Pop** | Mean | s.e. | Mean | s.e. | Mean | s.e. | Mean | s.e. | Mean | s.e. | Mean | s.e. | Mean | s.e. | Mean | s.e. | Mean | s.e. | Mean | s.e. |
| 5 | 0.3262 | 0.0215 | 6.2427 | 0.3864 | 12.7600 | 1.1956 | 0.0033 | 0.0006 | 19.5000 | 1.0473 | 7.0603 | 0.2864 | 31.4453 | 1.2220 | 0.8061 | 0.0753 | 175.2592 | 7.7111 | 0.7875 | 0.0085 |
| 6 | 0.3786 | 0.0248 | 8.0685 | 0.5281 | 7.8632 | 0.5987 | -0.0007 | 0.0005 | 18.3086 | 1.0018 | 8.0532 | 0.2911 | 25.9178 | 1.0601 | 0.7219 | 0.0750 | 155.5226 | 6.5056 | 0.7635 | 0.0113 |
| 119 | 0.2460 | 0.0183 | 8.5820 | 0.6516 | 7.4944 | 1.1135 | -0.0030 | 0.0006 | 15.3824 | 0.9459 | 7.3951 | 0.4302 | 24.8235 | 1.7065 | 0.4817 | 0.0542 | 147.7703 | 8.0031 | 0.7897 | 0.0093 |
| 121 | 0.2260 | 0.0180 | 11.2271 | 0.7128 | 8.3746 | 0.8150 | -0.0029 | 0.0005 | 19.1910 | 1.0245 | 9.1275 | 0.4025 | 29.0844 | 1.5049 | 0.4226 | 0.0448 | 133.4605 | 6.5735 | 0.7849 | 0.0074 |
| 133 | 0.4012 | 0.0215 | 6.9396 | 0.4609 | 10.9403 | 0.8921 | 0.0025 | 0.0006 | 18.4353 | 1.1175 | 6.5321 | 0.2654 | 28.9852 | 1.4189 | 0.7047 | 0.0682 | 168.4846 | 5.2470 | 0.7767 | 0.0071 |
| 136 | 0.2488 | 0.0181 | 6.9811 | 0.5609 | 6.4810 | 0.5979 | -0.0015 | 0.0006 | 13.9043 | 0.7684 | 6.6339 | 0.3239 | 22.4641 | 1.2637 | 0.4543 | 0.0553 | 153.0471 | 7.1239 | 0.7658 | 0.0084 |
| 139 | 0.3065 | 0.0235 | 5.0710 | 0.3364 | 4.5561 | 0.3681 | -0.0012 | 0.0007 | 17.5323 | 1.2317 | 5.3678 | 0.2420 | 24.1685 | 1.1650 | 0.5526 | 0.0692 | 175.8178 | 8.7406 | 0.7828 | 0.0084 |
| 250 | 0.3519 | 0.0242 | 8.2218 | 0.5651 | 7.9472 | 0.6484 | -0.0010 | 0.0005 | 17.4176 | 1.0584 | 6.9630 | 0.2557 | 26.1774 | 1.3242 | 0.6033 | 0.0613 | 152.1910 | 6.4447 | 0.7671 | 0.0091 |
| 255 | 0.3416 | 0.0209 | 5.9398 | 0.4185 | 9.3912 | 0.7563 | 0.0025 | 0.0006 | 17.6494 | 1.0529 | 6.5000 | 0.2619 | 28.3839 | 1.2499 | 0.6691 | 0.0824 | 149.5968 | 7.9760 | 0.7945 | 0.0074 |
| 302 | 0.3905 | 0.0215 | 12.2087 | 0.7733 | 9.1895 | 0.6842 | -0.0026 | 0.0004 | 17.0642 | 0.8971 | 8.6724 | 0.3471 | 25.5401 | 1.3041 | 0.5516 | 0.0824 | 145.7469 | 6.3107 | 0.7762 | 0.0091 |

**Table S6**. Results of the models testing for the effects of Fragment size, connectivity and their interaction on neutral genetic diversity indices. *A* average number of alleles per locus, *H_O_* observed heterozygosity, *H_E_* expected heterozygosity, *F_IS_* inbreeding coefficient. *F*-ratios and *P*-values are shown.

|  | **Fragment size** | | **Fragment connectivity** | | **Size * Conn.** | |
| --- | --- | --- | --- | --- | --- | --- |
|  | *F* | *P* | *F* | *P* | *F* | *P* |
| ***A*** | 1.7174 | 0.238 | 0.532 | 0.493 | 1.723 | 0.237 |
| ***H_O_*** | 0.944 | 0.085 | 4.238 | 0.085 | 0.907 | 0.378 |
| ***H_E_*** | 5.090 | 0.050 | 5.546 | 0.0567 | 4.064 | 0.0904 |
| ***F_IS_*** | 0.0760 | 0.792 | 0.296 | 0.606 | 0.004 | 0.954 |

**Table S7**. Results of the models testing for the effects of Fragment size, connectivity and their interaction on the percentage of phenotypic variance explained by differences among families (as a measure of genetically-based variance), for each trait. *F*-ratios and *P*-values are shown.

|  | **Fragment size** | | **Fragment connectivity** | | **Size * Conn.** | |
| --- | --- | --- | --- | --- | --- | --- |
|  | *F* | *P* | *F* | *P* | *F* | *P* |
| **Emergence rate** | 0.008 | 0.930 | 1.168 | 0.321 | 0.084 | 0.782 |
| **Size 1m** | 0.011 | 0.918 | 0.918 | 0.375 | 0.016 | 0.902 |
| **Size 6m** | 0.009 | 0.926 | 0.061 | 0.813 | 0.267 | 0.624 |
| **RGR** | 0.019 | 0.894 | 0.247 | 0.637 | 0.041 | 0.847 |
| **Seedling height** | 0.043 | 0.842 | 1.274 | 0.302 | 0.012 | 0.915 |
| **Leaf number** | 0.032 | 0.864 | 0.241 | 0.641 | 0.122 | 0.738 |
| **Leaf length** | 1.816 | 0.226 | 0.765 | 0.415 | 2.416 | 0.171 |
| **SLA** | 1.838 | 0.224 | 1.338 | 0.291 | 1.162 | 0.322 |
| **Leaf area** | 0.485 | 0.512 | 0.412 | 0.545 | 0.395 | 0.553 |
| **Chlorophyll fluorescence** | 0.222 | 0.654 | 0.046 | 0.838 | 0.337 | 0.583 |

**Table S8**. Results of the models testing for the effects of Fragment size, connectivity and their interaction on plasticity indices for each trait. *F*-ratios and *P*-values are shown.

|  | **Fragment size** | | **Fragment connectivity** | | **Size * Conn.** | |
| --- | --- | --- | --- | --- | --- | --- |
|  | *F* | *P* | *F* | *P* | *F* | *P* |
| **Plant height** | 0.022 | 0.887 | 0.000 | 0.998 | 0.006 | 0.942 |
| **SLA** | 2.004 | 0.206 | 0.232 | 0.647 | 2.354 | 0.176 |
| **Leaf area** | 1.581 | 0.255 | 0.103 | 0.760 | 0.576 | 0.476 |
| **Leaf number** | 0.296 | 0.606 | 0.188 | 0.679 | 0.233 | 0.646 |
| **Stomatal conductance** | 0.233 | 0.646 | 4.576 | 0.076 | 0.199 | 0.671 |
| **Chlorophyll fluorescence** | 1.211 | 0.313 | 0.796 | 0.407 | 1.118 | 0.330 |

**Table S9**. Correlations (Pearson’s *R* and *P*-value) between the percentage of phenotypic variance explained by differences among families (as a measure of genetically-based variance, top), and phenotypic plasticity (bottom), for each trait and population fitness.

|  | **Population Fitness** | |
| --- | --- | --- |
| **Neutral genetic variation** |  |  |
| *A* | \| -.3758 \| p=.285 \| \| --- \| --- \| | |
| *H_O_* | \| -.2351 \| p=.513 \| \| --- \| --- \| | |
| *H_E_* | \| .1831 \| p=.613 \| \| --- \| --- \| | |
| *F_IS_* | \| -.4919 \| p=.149 \| \| --- \| --- \| | |
|  |  | |
|  | **Population Fitness** | |
| **Genetic variation of** |  |  |
| Emergence rate | -.5153 | p=.127 |
| Size 1 m | .1361 | p=.708 |
| Size 6m | .4040 | p=.247 |
| RGR | .1320 | p=.716 |
| Plant height | .8874 | p=.001 |
| Leaf number | .2766 | p=.439 |
| Leaf length | .1559 | p=.667 |
| SLA | -.0120 | p=.974 |
| Leaf area | -.0684 | p=.851 |
| Chlorophyll fluorescence | -.5169 | p=.126 |
|  | **Population Fitness** | |
| **Plasticity of** |  |  |
| Plant height | -.3604 | p=.306 |
| Leaf number | -.2143 | p=.552 |
| Stomatal conductance | .2970 | p=.405 |
| SLA | -.3171 | p=.372 |
| Leaf area | .1660 | p=.647 |
| Chlorophyll fluorescence | -.3357 | p=.343 |

**Figure S1**. Soil water content (expressed as a percentage of field capacity) for plants in the well-watered and drought treatment. Percentage of field capacity was calculated based on weights of a random sample of 15 pots in each watering treatment. Measurements in the drought experiment were performed when drought pots contained soil at 40-50% of field capacity.


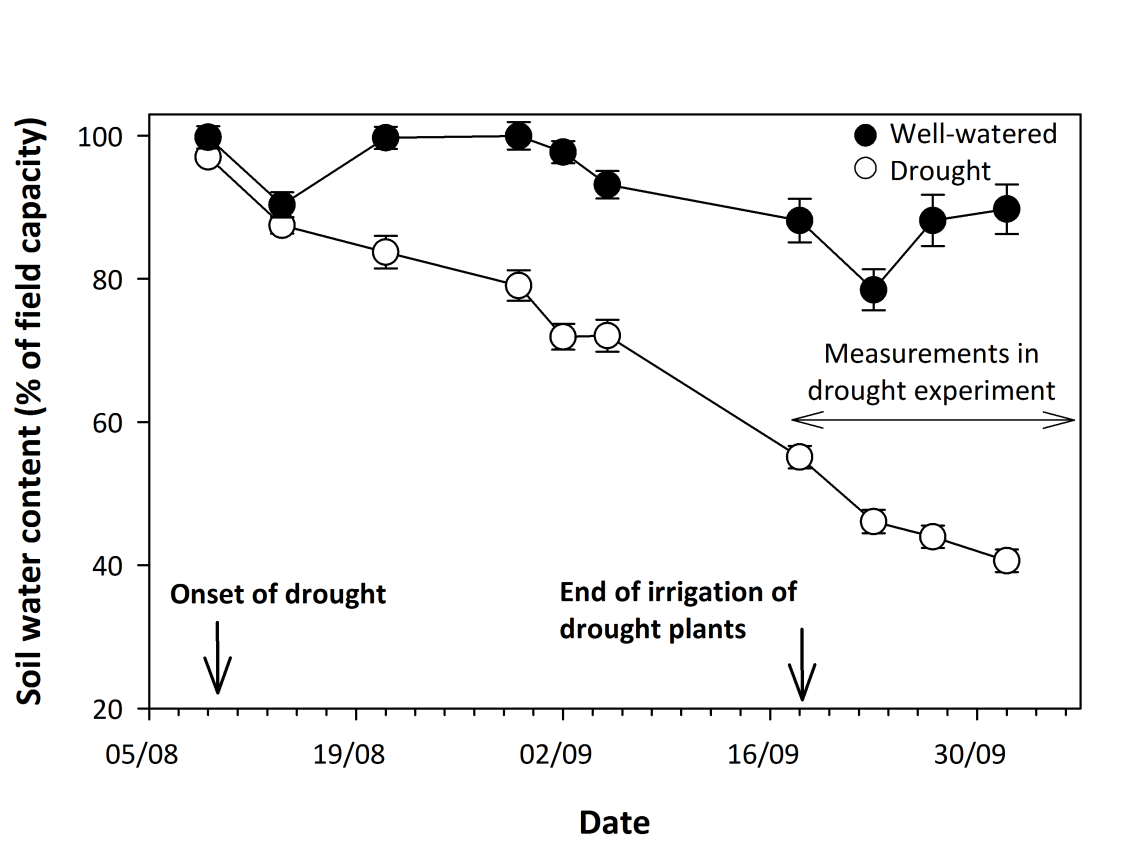


**Figure S2**. Percentage of phenotypic variance explained by differences among families within each population, for a set of key functional traits. This metric is used as an index of genetically-based differences, i.e., a proxy of evolutionary potential. Gray bars show significant genetic variation for a specific trait-population combination, based on individual models.





**Figure S3**. Survival curves for plants in the two contrasting watering treatments. Kaplan-Meier curves show cumulative survival at each timepoint. Curves were calculated based on ≈365 plants per treatment. The watering treatment exerted a significant effect on fitness (log-rank test).
